# Androstenedione response to recombinant human FSH is the most valid predictor of the number of selected follicles in polycystic ovarian syndrome: (a case-control study)

**DOI:** 10.1186/s13048-017-0330-7

**Published:** 2017-05-12

**Authors:** Eser Sefik Ozyurek, Tevfik Yoldemir, Gokhan Artar

**Affiliations:** 1 Bagcilar Research and Training Hospital Obgyn Department, Merkez Mh., Mimar Sinan Caddesi, 6. Sokak, 34100 Bagcilar, Istanbul, Turkey; 2 Marmara University Teaching and Research Hospital Obgyn Department, Fevzicakmak District Muhsin Yazicioglu Street 10 Ustkaynarca Pendik, Istanbul, Turkey

**Keywords:** Androgens, Androstenedione, Polycystic ovary syndrome, Ovulation induction, Folliculogenesis, Human FSH, Gonadotropins

## Abstract

**Background:**

We aimed to test the hypothesis that the correlation of the changes in the blood Androstenedione (A_4_) levels to the number of selected follicles during ovulation induction with low-dose recombinant human follicle stimulating hormone (rhFSH) is as strong as the correlation to changes in the blood Estradiol (E_2_) levels in polycystic ovary syndrome (PCOS).

**Methods:**

Prospective Case-control study conducted from October 2014 to January 2016. 61 non-PCOS control (Group I) and 46 PCOS (Group II) patients treated with the chronic low-dose step up protocosl with rhFSH. A_4_, E_2_, progesterone blood levels and follicular growth were monitored.. Univariate and hierarchical multivariable analysis were performed for age, BMI, HOMA-IR, A_4_ and E_2_ (with the number of selected follicles as the dependent variable in both groups). ROC analysis was performed to define threshold values for the significant determinants of the number of selected follicles to predict cyle cancellations due to excessive ovarian response.

**Results:**

The control group (Group I) was comprised of 61 cycles from a group of primary infertile non-PCOS patients, and the study group (Group II) of 46 cycles of PCOS patients. The analysis revealed that the strongest independent predictor of the total number of selected follicles in Group I was the E_2_(AUC) (B = 0.0006[0.0003-0.001]; *P* < 0.001); whereas for Group II, it was the A_4_ (AUC) (B = 0.114[0.04-0.25]; *P* = 0.01). Optimum thresholds for the A_4_ related parameters were defined to predict excessive response within Group II were 88.7%, 3.1 ng/mL and 5.4 ng*days for the percentage increase in A_4_, the maximum A_4_ value and area under the curve values for A_4_, respectively.

**Conclusion:**

A_4_ response to low-dose rhFSH in PCOS has a stronger association with the number of follicles selected than the E_2_ reponse. A_4_ response preceding the E_2_ response is essential for progressive follicle development. Monitoring A_4_ rather than E_2_ may be more preemptive to define the initial ovarian response and accurate titration of the rhFSH doses.

**Trial registration:**

The study was registered as a prospective case-control study in the ClinicalTrials.gov registry with the identifier NCT02329483.

## Background

Oligoovulation related to polycystic ovarian syndrome is treated with ovulation induction medications [1]. These patients have an increased risk of excessive ovarian response which is closely associated with the number of selected follicles [2]. Therefore, milder protocols have been developed [3]. In PCOS cases, the estradiol response to gonadotropin treatment is delayed and discordant with the visualized follicular responses [3]. Androstenedione (A_4_), mostly synthesized in the ovaries is a precursor of E_2_ [4, 5].

In this study, we aimed to test the hypothesis that the cumulative changes in A_4_ during ovulation induction with low dose rhFSH in PCOS cases are correlated to the number of selected follicles (follicles sized ≥12 mm) comparable to the the cumulative changes in E_2_.

## Methods

This is a prospective case-control study conducted between October 2014 and January 2016. Ethical permission was obtained from the Bagcilar Research and Training Hospital Research Ethics Committee. It was recorded as a prospective case-control study in the ClinicalTrials.gov registry with the identifier NCT02329483. The study was conducted in accordance with the Declaration of Helsinki. Informed consent for participation was obtained from all patients.

### Study setting

The study was conducted at the Bagcilar Research and Training Hospital Gynecology and Obstetrics Department, Infertility Section. Cycle monitoring was done with folliculometry with transvaginal sonography, E_2,_ P_4_ and A_4_ measurements.

### Study population

A total of 107 cycles of 61 Control-nonPCOS infertile (Group I) and 46 Study-PCOS infertile (Group II) women were included in the study. The study group was comprised of patients with anti-Müllerian hormone (AMH) levels ≥5 ng/mL (which is equivalent to PCOM (polycystic ovary morphology sonographically confirmed) [6–8]. PCOS was defined as the copresence of at least one of two of the following criteria combined with the PCOM; *(1): oligoamenorrhea-OA*: cycle length > 35 days, *(2): hyperandrogenism (HA)*: presence of clinical findings including hirsutism defined as the presence of coarse [long/pigmented] terminal hair over the most commonly encountered three or more regions within the Modified Ferriman-Gallwey Score System (the buttocks/perineum, sideburn, and neck areas which contributed greatly to the score in the geographic locale where this study was conducted) with or without elevated blood testosterone levels (>0.5 ng/mL) [7–12]. The control group was comprised of unexplained primary/secondary infertile women with AMH levels <5 ng/mL, not displaying any clinical findings associated with hyperandrogenism (HA) and with regular menstrual cycles. The inclusion criteria for both groups included ages within 20-35, with normal spermiograms or with mild male factor infertility (i.e.: male partners with only one the following abnormalities: sperm counts being lower than 20 million/ml *or* showing a normal morphology quotient of less than 4% *or* having a sperm motility lower than 40%; *AND* with post-wash total motile sperm counts equal to or higher than 5 million/ml), normal anatomic findings with the hysterosalpingography (no bilateral tubal obstruction or Müllerian anomalies), hormonally eugonadotropic, normal blood prolactin/thyroid-stimulating hormone (TSH) levels and being planned for controlled ovarian hyperstimulation and intrauterine insemination treatment. Exclusion criteria included: diabetes mellitus, BMI < 20 or >30, hypo or hypergonadotropism, other causes for hyperandrogenism, ≥2 abortions or ectopic pregnancy, additional medical disorders, ovarian cysts or previous pelvic surgery.

### Controlled ovarian hyperstimulation and intrauterine insemination

Ovulation induction was conducted with follitropin alpha (Gonal-f Multidose 450 IU; Merck-Serono-Turkey) starting with a dose of 37.5 U/day or 50 U/day as described by Homburg et al. [2]. Ovulation induction was started on the 3rd or the 4th day of a menstrual cycle having early cycle blood E_2_ levels <50 pg/mL and blood Progesterone levels <0.5 ng/mL and in the absence of any ovarian residual follicles larger than 15 mm to rule out the presence of a corpus luteum or any other cystic ovarian structure which could require further clarification. If a primary follicle response characterised by the appearance of a selected growing follicle of ≥10 mm and a rise ≥25% in blood E_2_ levels was not observed despite 14 days of rhFSH stimulation, the initial dose was increased initially to 75 U/day and +37.5 U/day, weekly at each additional incremental step (i.e. 112.5, 150 U/day). Blood E_2_, P_4_, and A_4_ levels were measured and follicle growth was monitored with transvaginal sonography at every visit (every 2-3 days) starting on day 2 or 3 of the cycles. Once 1 or 2 mature follicles ≥18 mm were observed, rhCG (Ovitrelle 150 μg; Merck-Serono-Turkey) s.c. was administered. Sperm washing and intrauterine insemination were carried within [36th–40th] hours. On the 15th day postinsemination, blood beta-hCG levels were measured and conception confirmed if the beta-hCG blood level was higher than 20mIU/mL.

### Cycle cancellation policy

Cycle cancellations were due to excessive ovarian responses (more than 2 selected follicles ≥16 mm or blood E_2_ level > 1500 pg/mL on the rhCG trigger day), no ovarian response despite dose step-up and stimulation for 28-30 days, or premature luteinisation (P_4_ blood level ≥ 1.3 ng/mL).

### Laboratory analysis of blood samples

Blood samples for hormone measurement were collected from the antecubital vein with a single puncture at every visit during ovulation induction. Samples were collected in a sterile tube and transferred to the lab on the same day. All except the A_4_ blood level measurement results were reported to the physicians on the afternoon of the same day. A_4_ blood levels were available 7-10 days later, and did not provide any guidance to management. A colorimetric ELISA assay (Abcam-USA; Kimera Istanbul-Turkey) was used to measure A_4_ levels. Measurements of AMH were made by using the AMH/MIS enzyme-linked immunosorbent assay. Testosterone values were assayed with the competitive immunoenzymatic colorimetric method. The serum FSH, luteinising hormone (LH), TSH, E_2_, and prolactin levels were measured using a chemiluminescent microparticle immunoassay.

### Data analysis

Univariate parametric tests were used for group comparisons. Significance was defined as a *P*-value <0.05. A_4_ (AUC) was calculated as the sum of the areas of trapezoids. Primary A_4_ response during ovulation induction was considered when a rise of ≥25% was observed in the basal A_4_ level. HOMA-IR (Homeostatic Model Assessment of Insulin Resistance) Index was calculated for each patient by using an online calculator.

Hierarchial multivariable regression analysis was conducted in both groups to study in a three level linear regression model the contribution of *three sets of independent variables* including the (Model 1) age, BMI and HOMA-IR variables; (Model 2) the E_2_(AUC) values and (Model 3) (the androstenedione related variables: the primary blood androstenedione level and the A_4_ (AUC) values); stepwise, defining *changes in R* ^*2*^ *values (ΔR* ^*2*^) representing the additional effect of each of these newly added independent variable sets, on *the total number of selected follicles*. The SPSS 20.0 and Microsoft Excel 2010 were used.

ROC analysis was performed to define threshold values of the significant determinants of the number of selected follicles to predict cyle cancellations due to excessive ovarian response.

## Results

A total of 107 cycles of infertile women Group I: 61 non-PCOS and Group II: 46 PCOS were followed in 16-months. The PCOS phenotypes were: PCOM (polycystic ovarian morphology:AMH ≥ 5 ng/mL)/OA (oligo/amenorrhea): 4 patients; PCOM/HA (Hyperandrogenism): 27 patients; PCOM/HA/OA: 15 patients. Mild male factor infertility was present in (22/61) 36% of Group I and (15/46) 32.6% in Group II (*P* = 0.5). The HOMA-IR score was ≥4.5 in 6/61 (9,8%) and 9/46 (19,6%) of patients in Groups I and II, respectively. The patient characteristics are summarised in (Table 1). There were 6/61 (9.8%) and 9/46 (19.5%) conceptions in Groups I and II, respectively. The comparison of hormonal characteristics among the conceived and the nonconceived subjects within Groups I and II are summarized in (Table 2). No conception was achieved in the absence of a primary androstenedione response earlier than the primary estradiol response. Cancelled cycles were not included in this comparison.

**Table 1:** Cycles analysed in this study

	Group I	Group II
	(Control Group)	(PCOS Group)
Age	29.8 ± 0.6	28.7 ± 0.6
Duration of infertility (years)	3.5 ± 0.3	3.4 ± 0.4
BMI (kg/m^2^)^α^	25 ± 0.7	27.7 ± 0.6
AMH(ng/mL)^α^	2.4 ± 0.2	9 ± 0.9
HOMA^α^	2.8 ± 0.4	3.8 ± 0.4
FSH (mIU/ml)^α^	7.2 ± 0.3	6.3 ± 0.2
LH (mIU/ml)^α^	6.5 ± 0.5	9.8 ± 0.6
TSH (μIU/mL)	2.7 ± 0.2	3.3 ± 0.7
PRL (ng/ml)	20.9 ± 1.2	22 ± 1.9
Initial Dose (IU/day)	70 ± 4.1	65 ± 3.7
Cycle Length (days)	12.2 ± 4.1	17 ± 0.6
Primary follicular/E_2_ response day^α^	6.7 ± 0.4	9.4 ± 0.4
EM at trigger day (mm)	9.9 ± 0.3	9.4 ± 0.3
Maximum E_2_ (pg/dl)	453 ± 42.1	556.9 ± 89.3
Follicles >16 mm (n)	1.1 ± 0.1	1.4 ± 0.2
Follicles 12-16 mm (n)	1.2 ± 0.2	1.6 ± 0.3
Total number of follicles	1.9 ± 0.2	2.2 ± 0.3
P_4_ at trigger day (ng/mL)	0.7 ± 0.1	0.8 ± 0.1
Day 3 Total Testosterone (ng/mL)^α^	0.34 ± 0.28	0.87 ± 0.3
Primary androstenedione level (ng/mL)^α^	0.9 ± 0.1	1.4 ± 0.1
Primary androstenedione respond day^α^	6.2 ± 0.3	7.4 ± 0.4
Time Lag (A_4_ → E_2_) initial responses (days)^α^	0.5 ± 0.4	2 ± 0.5
Maximum A_4_(ng/mL)^α^	1.8 ± 0.1	2.6 ± 0.3
Rise in A_4_ (%)^α^	66.5 ± 6.9	84.1 ± 7.4
A_4_ (AUC) (ng*days)^α^	3.6 ± 0.3	5.5 ± 1.3
A_4_ on the trigger day (ng/mL)^α^	1.3 ± 0.1	2.5 ± 0.4

*PCOS* polycystic ovarian syndrome, *AMH* Anti-Müllerian hormone, *HOMA* homeostasis model for assesment of insulin resistance, *FSH* follicle-stimulating hormone, *LH* luteinising hormone, *TSH* thyroid-stimulating hormone, *E*_*2*_ estradiol, *PRL* prolactin, *EM* endometrial thickness, *P*_*4*_ progesterone, *AUC* area under the curve, *A*_*4*_ androstenedione

^α^ *P* < 0.05

**Table 2:** Comparison of the hormonal characteristics among the conceived and nonconceived subjects

	Group I (Control Group)		Group II (PCOS Group)	
	Did not conceive	Conceived	Did not conceive	Conceived
Primary A_4_^a^	0,88 ± 0.45	0,96 ± 0.2	1,39 ± 0.08	1.4 ± 0.33
A_4_ (AUC)^b^	3,06 ± 2.8	2.8 ± 1.3	4,2 ± 0.7	10.7 ± 7.1
E_2_(AUC)^c^	1916,6 ± 206.3	2942.7 ± 1058.4	2654,2 ± 677.3	4212.7 ± 767.2
AMH^d^	2,53 ± 1.2	3.0 ± 0.41	9.23 ± 1	8.6 ± 1.5
HOMA-IR^e^	3,08 ± 0.3	2.4 ± 0.6	3,2 ± 0.34	2.22 ± 0.33
*Time Lag A*_*4*_ *-E*_*2*_ *Response*	−0,92 ± 0.46	−0,67 ± 0.4	−0,23 ± 0.11^af^	−3.79 ± 0.86^af^

^a^The initial blood androstenedione level (ng/mL)

^b^Area under the curve value for Androstenedione (ng*days)

^c^Area under the curve value for estradiol (pg*days)

^d^Antimüllerian hormone (ng/mL)

^e^Homeostasis Model for Assessment of Insulin Resistance

^f^ *P*<0.05

Nineteen cycles were cancelled due to excessive response (*n* = 8), no response (*n* = 6), or premature luteinisation (*n* = 5). In those cycles cancelled due to no response, there was no primary A_4_ response.

Univariate analysis revealed that the primary, maximum, and AUC values for A_4_ and the primary blood testosterone levels were all higher in Group II than those in Group I (Table 1).

Plateauing or decreasing A_4_ levels before the trigger day were observed in 33/61 (54,1%) of completed cycles in Group I and 14/46 (30,4%) of cycles in Group II. None of these made any significant difference in the basic characteristics or outcome parameters.

**Fig. 1:**
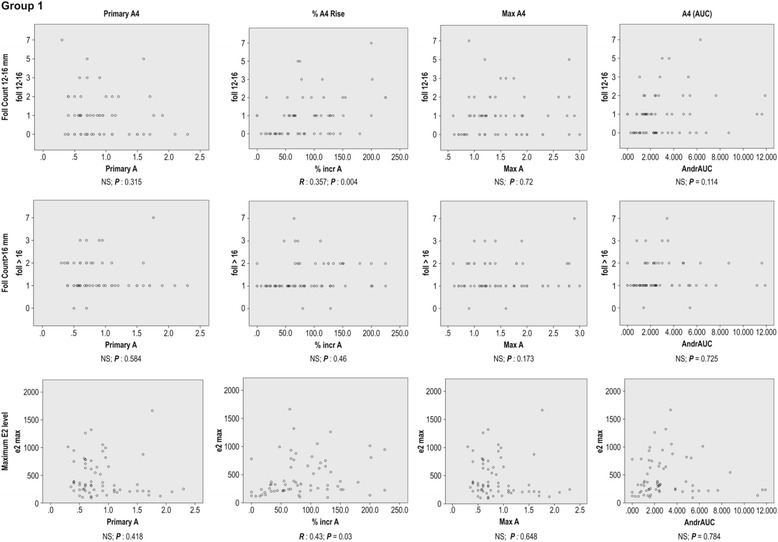
Correlation of A_4_ related parameters with cycle outcome in Group I

**Fig. 2:**
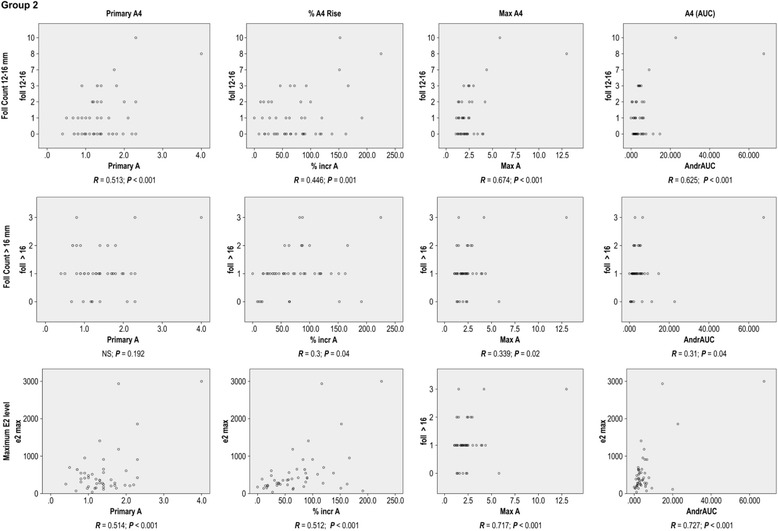
Correlation of A_4_ related parameters with cycle outcome in Group II

The correlation of A_4_ parameters with the selected follicle numbers and E_2_ (AUC) values are summarised in Figs. (1 and 2; and Tables (3 and 4): The A_4_(AUC) was correlated with the number of selected follicles and E_2_(AUC) in Group II, but not correlated with any one of these parameters in Group I The primary A_4_ levels were correlated to the primary testosterone levels in both Groups I and II.

**Table 3:** Correlation of A_4_ parameters with cycle outcome parameters in Group I (Control Group)

*R* ^*a*^	Primary A_4_	increase A_4_%^b^	Max A_4_	A_4_ AUC
Foll 12-16^d^	NS	0.36^γ^	NS	NS
Foll >16^c^	NS	NS	NS	NS
Maximum E_2_	NS	0.43^γ^	NS	NS
*Total Foll* ^e^ *Selected*	*NS*	*0.32* ^*γ*^	*NS*	*NS*

E_2_ = Estradiol; A_4_ = Androstenedione; AUC = Area under the curve; Foll = Number of follicles

^γ^ *P* < 0.005

^a^Pearson Correlation constant

^b^Percentage rise in the A_4_ level

^c^number of follicles >16 mm

^d^number of follicles 12-16 mm

^e^number of follicles >12 mm

**Table 4:** Correlation of A_4_ parameters to the cycle outcome parameters in Group II (PCOS Group)

*R* ^a^	Primary A_4_	increase A_4_%^b^	Max A_4_	A_4_ AUC
Foll 12-16^d^	0.513^γ^	0.446^γ^	0.674^γ^	0.625^γ^
Foll >16^c^	NS	0.3	0.339^γ^	0.31^γ^
Maximum E_2_	0.514^γ^	0.512^γ^	0.717^γ^	0.727^γ^
*Total Foll Selected* ^e^	*0.542* ^*γ*^	*0.5* ^*γ*^	*0.73* ^*γ*^	*0.673* ^*γ*^

E_2_ = Estradiol, A_4_ = Androstenedione, AUC = Area under the curve, Foll = Number of follicles

^a^Pearson Correlation constant

^b^Percentage rise in the A_4_ level

^c^number of follicles >16 mm

^d^number of follicles 12-16 mm

^e^number of follicles >12 mm

^γ^A significant correlation; *P*<0.05

*Hierarchical multivariable regression analysis* was conducted seperately for *Groups I and II*. The findings of the analysis are summarized in Tables 5(a,b) and 6(a,b). The total number of selected follicles (≥12 mm) was the dependent (outcome) variable. The effect of age, BMI and HOMA-IR on the total number of selected follicles were not significant in either Group I or II. In Group I: the estradiol (AUC) was *the strongest independent factor* (B = 0.0006[0.0003-0.001]; *P* < 0.001), *whereas* in Group II: the A_4_(AUC) (B = 0.114 [0.04-0.25]; *P* = 0.01) was *the strongest independent factor effecting the total number of selected follicles.*

**Table 5:** Hierarchical multivariable regression analysis of independent variables in Group I (Control Group)

Model Summary for Group I				
(A): Model Summary				
Models		Change Statistics		
	R Square	R Square Change	F Change	Sig. F Change
1^a^	0,07	0,07	1,1	NS
2^b^	***0,42***	***0,35***	***25,4***	***<0.001***
3^c^	0,44	0,01	0,5	NS
(B): Coefficients				
Models		Coefficients		
		B	Std. Error	P
1^a^	(Constant)	3,49	1,78	0,06
	Age	0,0004	0,05	0,99
	BMI	−0,02	0,04	0,59
	HOMA-IR	−0,19	0,13	0,15
2^b^	(Constant)	−0,46	1,63	0,78
	Age	0,06	0,04	0,17
	BMI	−0,01	0,03	0,75
	HOMA-IR	0,03	0,11	0,76
	*E*_*2*_ *(AUC)*	***0,0006***	***0,0001***	***<0.001***
3^c^	(Constant)	−0,50	1,65	0,77
	Age	0,06	0,04	0,20
	BMI	−0,02	0,03	0,66
	HOMA-IR	−0,03	0,13	0,83
	E_2_ (AUC)	***0,0006***	***0,0001***	***<0.001***
	Primary A	0,48	0,49	0.68
	A_4_(AUC)	0,01	0,07	0,85

^a^Model 1: including the independent variables Age, BMI, HOMA-IR

^b^Model 2: adding the independent variable E2 (AUC) to the previous Model 1

^c^Model: adding the independent variables Primary A and A4(AUC) to the previous Model 2

**Table 6:** Hierarchical multivariable regression analysis of independent variables in Group II (PCOS Group)

Model Summary for Group 2				
(A): Model Summary				
Models		Change Statistics		
	R Square	R Square Change	F Change	Sig. F Change
1^a^	0,05	0,05	0,3	0.8
2^b^	***0,49***	***0,44***	***16,6***	***<0.001***
3^c^	***0,63***	***0,14***	***15,1***	***0.001***
(B): Coefficients				
Models		Coefficients		
		B	Std. Error	P
1^a^	(Constant)	5,68	3,37	0,10
	Age	−0,05	0,08	0,57
	BMI	−0,07	0,10	0,50
	HOMA-IR	−0,10	0,21	0,64
2^b^	(Constant)	6,14	2,52	0,02
	Age	−0,06	0,06	0,36
	BMI	−0,12	0,08	0,13
	HOMA-IR	−0,08	0,15	0,62
	*E*_*2*_ *(AUC)*	***0,002***	***0,001***	***<0,01***
3^c^	(Constant)	4,62	2,43	0,07
	Age	−0,01	0,06	0,89
	BMI	−0,14	0,07	0,05
	HOMA-IR	−0,06	0,14	0,67
	E_2_ (AUC)	0,0005	0,0003	0,34
	Primary A	0,57	0,60	0,35
	A_4_(AUC)	***0,114***	***0,03***	***0.01***

^a^Model 1: including the independent variables Age, BMI, HOMA-IR

^b^Model 2: adding the independent variable E2 (AUC) to the previous Model 1

^c^Model: adding the independent variables Primary A and A4(AUC) to the previous Model 2

Optimum thresholds for the A_4_ related parameters were defined to predict excessive response within Group II combined were as in Table 7: 88.7%, 3.1 ng/mL and 5.4 ng*days for the percentage increase in A_4_, the maximum A_4_ value and area under the curve values for A_4_, respectively.

**Table 7:** ROC analysis of Androstenedione related parameters (maximum A4, percentage increase in A4 and A4 area under the curve) to define optimum threshold parameters to predict cycle cancellations in Group II

	Area Under Curve ± SEM [5-95p]	Optimum Threshold	Sensitivity (%)	Specificity (%)
A_4_ percentage increase	0.73 ± 0.07 [0.6-0.87]	88.7%	75	72
Maximum A_4_	0.78 ± 0.19 [0.66-0.9]	3.1 ng/mL	75	74
A_4_ (AUC)	0.78 ± 0.06 [0.67-0.9]	5.4 ng*day	70	72

## Discussion

In this study, we observed that in PCOS, the cumulative A_4_ response to low-dose rhFSH is a more valid measure of the number of selected follicles than the cumulative Estradiol (E_2_) response [13]._._ The early and midfollicular A_4_ variations are more critical determinants than the late follicular variations (following follicle selection), because drops or plateauing observed in A_4_ in the late stages did not influence cycle outcome. The A_4_ respond to rhFSH was earlier than that of E_2_ in cycles with progressive follicular growth and conception.

In six cycles cancelled due to lack of response to rhFSH, there was no A_4_ response. In contrast, in four of the eight cycles cancelled due to excessive response, a dosage step-up had been made due to lack of E_2_response, while there had already been an initial A_4_response. If this corrective information could have been taken into account, an unnecessary step-up could have been avoided.

PCOS is the most common cause of anovulatory infertility, and is reported to comprise 15.3% of the women living in the geographic region where this study was conducted [14]. PCOS is characterised by three main elements: follicular growth arrest, hyperandrogenism and excessive folliculogenesis [15].

The chronic low-dose step up protocol used for ovulation induction in PCOS patients requires up to 14 days of rhFSH treatment to overcome a temporary ovarian refractoriness ending with follicle selection [16]. The follicular growth response initiated by rhFSH in the granulosa cell component when treated with rhFSH is propagated to the theca cell component. During this initial response, in PCOS patients, the reversal of the FSH/LH effect in favour of FSH and aromatisation may not be as hormonally evident with rising E_2_ blood levels as the thecal androgen response, especially at earlier stages. Granulosa cells of the antral follicle at this stage normally respond to this early rise in A_4_ by increasing their aromatase activities, which is strongly counteracted by high AMH levels in the follicular microenvironment of the PCOS follicles, analogous to the AMH-FSH counteraction at later stages of follicular growth [17]. Thus, the transient follicular growth arrest is observed at this early stage due to the AMH-androgen counteraction [18, 19]. High androgen and AMH concentrations also contribute to the microenvironment that fosters excessive folliculogenesis and follicular growth arrest [11, 20–23].

Basal androgen blood levels measured at the beginning or during induction cycles have been reported in various studies in low responders, patients with diminished ovarian reserves, or normal responders, but not in PCOS cases. Ferrario et al. showed in a group of older women with low response that A_4_ levels measured at the beginning of IVF cycles were predictors of positive outcome [24]. Similarly, Sun et al. have shown in a study of 1413 infertile women going through their first cycle of IVF that the testosterone blood levels measured at the beginning of the treatment cycles were predictors of the number of follicles larger than 14 mm on the day of hCG trigger, but were not predictors of conception [25]. It would be interesting and supplementary to monitor the testosterone response to rhFSH during ovulation induction cycles in prospective observational studies.

Our study had some *potential causes of bias and limitations* which need to be addressed. From the perspective of the Rotterdam 2003 definition of PCOS, cases with the nonPCOM/HA/OA (defined as phenotype B) may be being underrepresented in the infertile PCOS patients group (Group II) [26]. Another limitation was that the BMI’s in *Group II* were slightly, but significantly higher than *Group I*. However, in the hierarchical multivariable regression conducted, BMI as well as age and HOMA-IR were included in a separate model and their effects on the total number of selected follicles were found to be insignificant in both *Groups I and II*.

## Conclusion

The findings in this clinical study suggest that the reactive rise in androstenedione in the early follicular phase is a better predictor of the number of follicles selected than the conventionally used reactive rise in estradiol in PCOS cases. The longer/higher is the increase in its blood levels, the more are the follicles joining the growing cohort with an increasing risk of excessive ovarian response. On the other hand, androstenedione is an earlier and more reliable marker of the initial ovarian response to gonadotropins and this earlier response may be essential for progressive follicle growth and possibly a conception in an ovulation induction and intrauterine insemination cycle (using rhFSH) in PCOS. It still needs to be further studied in prospective studies encompassing induction cycles managed mainly with A_4_ monitoring, to provide stronger evidence if androstenedione monitoring provides a more valid and useful information to indicate ovarian response and if an earlier androstenedione response is associated with conception.

## Abbreviations

A4: Androstenedione
AMH: Antimüllerian hormone
AUC: Area under the curve
beta-hCG: beta human chorionic gonadotroopin
BMI: Body mass index
E2: Estradiol.
FSH: Follicle stimulating hormone
HA: Hyperandrogenism
HOMA-IR: Homeostatic model assessment of insulin resistance
IVF: In vitro fertilization
OA: Oligo/amenorrhea
P4: Progesterone
PCOM: polycystic ovarian morphology
PCOS: Polycystic ovary syndrome
rhFSH: Recombinant human follicle stimulating hormone
ROC: Receiver operating curve
TSH: Thryoid stimulating hormone

## References

[1] Homburg R, Howles CM (1999). Low-dose FSH therapy for anovulatory infertility associated with polycystic ovary syndrome: rationale, results, reflections and refinements. Hum Reprod Update.

[2] Nastri CO, Teixeira DM, Moroni RM, Leitão VM, Martins WP (2015). Ovarian hyperstimulation syndrome: pathophysiology, staging, prediction and prevention. Ultrasound Obstet Gynecol.

[3] White DM, Polson DW, Kiddy D, Sagle P, Watson H, Gilling-Smith C (1996). Induction of ovulation with low-dose gonadotropins in polycystic ovary syndrome: an analysis of 109 pregnancies in 225 women. J Clin Endocrinol Metab.

[4] Tsang BK, Taheri A, Ainsworth L, Downey BR (1987). Secretion of 17 alpha-hydroxyprogesterone, androstenedione, and estrogens by porcine granulosa and theca interna cells in culture. Can J Physiol Pharmacol.

[5] Arai S, Ito K (2010). Androstenedione. Nihon Rinsho.

[6] Dewailly D, Lujan ME, Carmina E, Cedars MI, Laven J, Norman RJ (2014). Definition and significance of polycystic ovarian morphology: a task force report from the androgen excess and polycystic ovary syndrome society. Hum Reprod Update.

[7] Catteau-Jonard S, Dewailly D (2011). Anti-Mullerian hormone and polycystic ovary syndrome. Gynecol Obstet Fertil.

[8] Dewailly D, Pigny P, Soudan B, Catteau-Jonard S, Decanter C, Poncelet E, Duhamel A (2010). Reconciling the definitions of polycystic ovary syndrome: the ovarian follicle number and serum anti-Müllerian hormone concentrations aggregate with the markers of hyperandrogenism. J Clin Endocrinol Metab.

[9] Hassa H, Tanir HM, Yildirim A, Senses T, Eskalen M, Mutlu FS (2005). The hirsutism scoring system should be population specific. Fertil Steril.

[10] Yildiz BO, Bolour S, Woods K, Moore A, Azziz R (2010). Visually scoring hirsutism. Hum Reprod Update.

[11] Pasquali R, Zanotti L, Fanelli F, Mezzullo M, Fazzini A (2016). Defining Hyperandrogenism in women with polycystic ovary syndrome: a challenging perspective. J Clin Endocrinol Metab.

[12] Romualdi D, Di Florio C, Tagliaferri V, De Cicco S, Gagliano D, Immediata V (2016). The role of anti-Müllerian hormone in the characterization of the different polycystic ovary syndrome phenotypes. Reprod Sci.

[13] Azziz R, Carmina E, Dewailly D, Diamanti-Kandarakis E, EscobarMorreale HF, Futterweit W (2006). Positions statement: criteria for defining polycystic ovary syndrome as a predominantly hyperandrogenic syndrome: an androgen excess society guideline. J Clin Endocrinol Metab.

[14] Yildiz BO, Bozdag G, Yapici Z, Esinler I, Yarali H (2012). Prevalence, phenotype and cardiometabolic risk of polycystic ovary syndrome under different diagnostic criteria. Hum Reprod.

[15] Jonard S, Dewailly D (2004). The follicular excess in polycystic ovaries, due to intra-ovarian hyperandrogenism, may be the main culprit for the follicular arrest. Hum Reprod Update.

[16] Brown JB (1978). Pituitary control of ovarian function--concepts derived from gonadotrophin therapy. Aust N Z J Obstet Gynaecol.

[17] Almahbobi G, Anderiesz C, Hutchinson P, McFarlane JR, Wood C, Trounson AO (1996). Functional integrity of granulosa cells from polycystic ovaries. Clin Endocrinol.

[18] Lenie S, Smitz J (2009). Functional AR signaling is evident in an in vitro mouse follicle culture bioassay that encompasses most stages of folliculogenesis. Biol Reprod.

[19] Li M, Schatten H, Sun QY (2009). Androgen receptor's destiny in mammalian oocytes: a new hypothesis. Mol Hum Reprod.

[20] Weil SJ, Vendola K, Zhou J, Adesanya OO, Wang J, Okafor J (1998). Androgen receptor gene expression in the primate ovary: cellular localization, regulation, and functional correlations. J Clin Endocrinol Metab.

[21] Pache TD, Fauser BC (1993). Polycystic ovaries in female-to-male transsexuals. Clin Endocrinol.

[22] Murray AA, Gosden RG, Allison V, Spears N (1998). Effect of androgens on the development of mouse follicles growing in vitro. J Reprod Fertil.

[23] Magarelli PC, Zachow RJ, Magoffin DA (1996). Developmental and hormonal regulation of rat theca-cell differentiation factor secretion in ovarian follicles. Biol Reprod.

[24] Ferrario M, Secomandi R, Cappato M, Galbignani E, Frigerio L, Arnoldi M (2015). Ovarian and adrenal androgens may be useful markers to predict oocyte competence and embryo development in older women. Gynecol Endocrinol.

[25] Sun B, Wang F, Sun J, Yu W, Sun Y (2014). Basal serum testosterone levels correlate with ovarian response but do not predict pregnancy outcome in non-PCOS women undergoing IVF. J Assist Reprod Genet.

[26] Rotterdam ESHRE/ASRM-Sponsored PCOS Consensus Workshop Group (2004). Revised 2003 consensus on diagnostic criteria and long-term health risks related to polycystic ovary syndrome. Fertil Steril.

